# Assessment of Accessory Canals and Foramina in the Mandibular Arch Using Cone-Beam Computed Tomography and a New Classification for Mandibular Accessory Canals

**DOI:** 10.1155/2022/5542030

**Published:** 2022-02-14

**Authors:** Pooja Muley, Lata Kale, Sneha Choudhary, Sami Aldhuwayhi, Amar Thakare, Sreekanth Kumar Mallineni

**Affiliations:** ^1^, Consultant Oral and Maxillofacial Radiologist, Aurangabad, Maharashtra, India; ^2^Department of Oral Medicine and Radiology, CSMSS Dental College and Hospital, Aurangabad, Maharashtra, India; ^3^Department of Oral Medicine and Radiology, Teerthanker Mahaveer Dental College and Research Centre, Moradabad, Uttar Pradesh, India; ^4^Department of Prosthodontics, College of Dentistry, Majmaah University, Al-Majmaah 11952, Saudi Arabia; ^5^Department of Preventive Dental Science, College of Dentistry, Majmaah University, Al-Majmaah 11952, Saudi Arabia; ^6^Center for Transdisciplinary Research (CFTR), Saveetha Institute of Medical and Technical Sciences, Saveetha Dental College, Saveetha University, Chennai 600077, Tamil Nadu, India

## Abstract

**Objectives:**

The objectives of the study include the following: (i) to assess the presence of accessory canals and foramina in the body of the mandible using cone-beam computed tomography (CBCT), (ii) to evaluate the location, number, diameter, and length of accessory canals in the body of the mandible, and (iii) to propose a new classification for mandibular accessory canals based on the location.

**Methods:**

A total of 50 (25 males and 25 females) CBCT scans were analyzed in three anatomical planes and three-dimensional (3D) reconstructions for the exact number, location, diameter, and accessory length canals and accessory foramina in the body of the mandible. The statistical analysis used was an independent *t*-test.

**Results:**

Out of 50 CBCT scans, a total of 243 accessory canals and 245 accessory foramina were found. About 53% of accessory canals and foramina were found in males, while 47% were observed in females; 89% were evident in the anterior region, and only 11% were in the posterior region. The majority (64%) of the medial lingual canals had a diameter greater than or equal to 1 mm, while only 32% of accessory canals had a diameter of less than 1 mm (*p* < 0.05). The mean length of median lingual canals in females was 1.2910 ± 0.2582 mm and 2.6438 ± 0.5288 mm in male subjects. Mandibular accessory canals are classified broadly into anterior and posterior accessory canals, which have further subdivisions.

**Conclusion:**

CBCT plays a dynamic role in determining the mandible location of various neurovascular structures, including accessory canals and foramina. Female subjects were observed with more accessory canals and foramina and more common in the anterior region than in the posterior region.

## 1. Introduction

In the recent era, we are residing in an era of implants, and hence, it becomes essential to have in-depth knowledge and understanding of the anatomy of neurovasculature and its variations [1–4]. Accessory canals in the mandibular arch are attributed to the incomplete fusion nerve branches supplying the teeth [4]. These anatomical structures were proposed as explanations for the failure of local anesthetic blocks [5]. Accessory or nutrient canals are the canals other than the mandibular canal, running through the buccal and/or lingual cortical plates of the mandible into the trabecular bone [2–4]. Radiographic examinations are used for assessing the location, relation, and dimension of these accessory canals [2, 3, 5]. Conventional radiographs only provide two-dimensional images of the three-dimensional structures [5]. Compared to conventional two-dimensional imaging, cone-beam computed tomography (CBCT), a three-dimensional imaging technique, has increased accuracy, a higher resolution, reduced scan time, reduced radiation dose, and reduced cost for the patient. It also eliminates the superimposition of the surrounding structures, providing additional clinically relevant information [5], thus emerging as the most suitable technique to diagnose the presence of accessory canals. The majority of the studies [6–14] reported in the literature that has radiographically assessed the accessory canals have used panoramic radiographs. Anatomical variations in dimensions and morphology of the mandible accessory canals remain not extensively documented. Thus, a detailed study of all the anatomical variations in mandibular neurovascularization becomes necessary. Hence, the present study was aimed at evaluating the presence and number of accessory canals and foramina radiographically in the mandible body using CBCT and assessing the location, length, and diameter of the accessory canals and foramina in the mandible.

## 2. Materials and Method

The present observational study was a collaborative study in the Department of Oral Medicine and Radiology, CSMSS Dental College and Hospital, Aurangabad, Maharashtra, India, and the Department of Prosthodontics, College of Dentistry, Majmaah University (MUREC-NOV.08/COM-2020/8-4). Ethical clearance was obtained from the institutional ethical committee for the present study. Healthy patients in the age range of 20-50 years without any signs of systemic disease and dental anomalies involving the mandible were included in the study. Pregnant patients, completely edentulous patients, and patients with a history of maxillofacial trauma, craniofacial malformations, or syndromes were excluded from the study. All the selected patients were informed about the study in detail, and written consent was obtained prior to the study. The case history was recorded briefly, and CBCT imaging of the whole mandible was considered for the study purpose. All the necessary patient protection measures were undertaken during CBCT exposure.

### 2.1. CBCT Imaging and Analysis (Figure 1)

The three-dimensional imaging data were acquired at 87 kV and 8 mA. The scan time was within a range of 8.01 to 8.655 seconds, and the voxel size was 180 *μ*m with 0.180 mm slice thickness of the CBCT images. Scans were obtained in sagittal, axial, and coronal planes, and each multiplanar data measuring 180 × 180 × 180 *μ*m pixels at 16 bits was stored in the computer. This patient data was exported in DICOM (Digital Imaging and Communications in Medicine) format by using the digital medical imaging equipment, which was then studied for the detailed analysis of the accessory canals using CS 3D software. The density and the contrast of images were optimally adjusted so that the accessory canals were clearly visualized. The image analysis was done using curved slicing mode, and all the interpretations and assessments were made in a cross-sectional view. The exact location of the accessory canals was identified and traced. The tracing of the accessory canals and the length of canals were measured in the cross-sectional views, taking the sagittal section as a guide since the canals were best visualized in this view. The canals' diameter was measured in the cross-sectional views at the exit of canals, and in the case of bifid canals, a canal with a greater diameter was considered.

The study was aimed at assessing the presence of accessory canals and foramen in the mandible keeping in mind the risk involved in any of these least explored and poorly understood anatomical structures getting injured during surgical procedures or implant placement procedures. The mandible was divided into anterior and posterior sections, taking mental foramina as a reference point for ease of study. The anterior section was defined as “part of the mandible between two mental foramina,” and the posterior section was defined as “part of the body of the mandible posterior to the mental foramina till the retromolar fossa region.” The canals arising in the midline of the mandible were named median lingual canals. The measurements were done using the measurement tool in the CS 3D software. The mandibular canal was not considered for evaluation. As implant placement is usually done anterior to the retromolar fossa region, we have not included the ascending ramus and total length of the mandibular canal in the study. The CBCT scans were done in the study for those who were planned for implant placement; we tried to expose the patients to the least possible radiation while gathering the necessary information required for the study.

### 2.2. Statistical Analysis

The data thus collected were tabulated and subjected to statistical analysis. The statistical analysis was performed using the Statistical Package for the Social Sciences (SPSS, version 17.0, Illinois, Chicago, USA, for MS Windows). The statistical significance of the difference in the distribution and measurements was tested using an independent *t*-test after confirming the underlying normality assumption. The *p* value was considered at *p* < 0.05 significance with a 95% confidence interval.

## 3. Results

The present study results showed that at least one canal and foramina were present in all the scans. Fifty healthy patients (25 males and 25 females) between the ages of 20 and 50 visiting the routine outpatient department were included. A total of 243 accessory canals and 245 accessory foramina were found, out of which 53% accessory canals were found in males while 47% were seen in females.

### 3.1. Accessory Canals and New Classification

**Figure 2:**
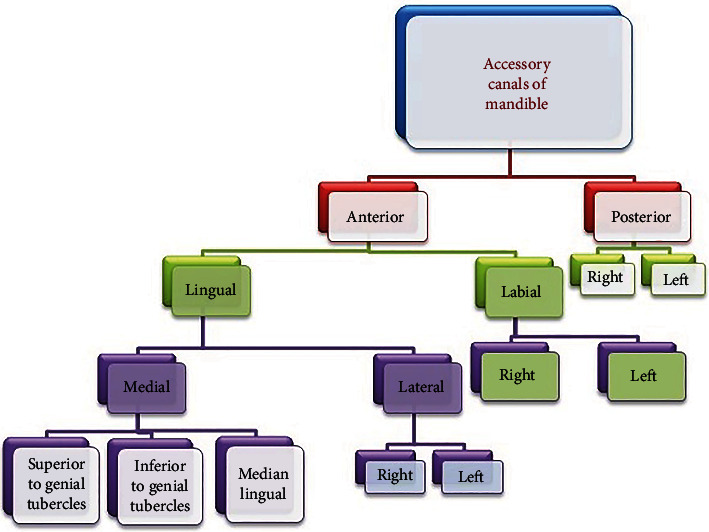
Schematic representation of the proposed classification of the accessory canals of the mandible.

**Figure 3:**
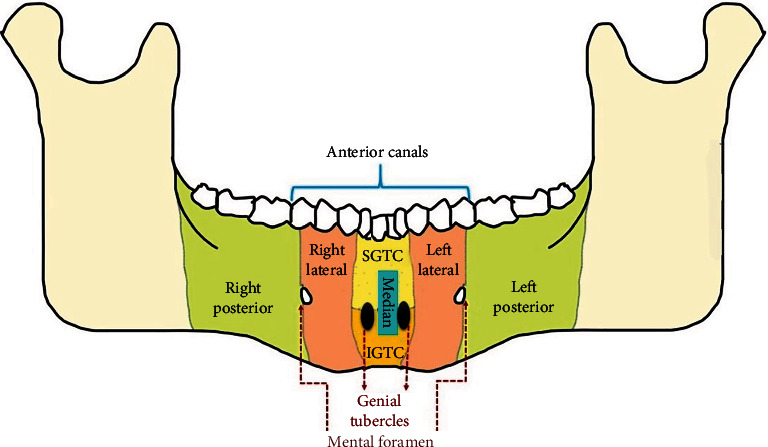
Schematic representation of the accessory canals in the mandibular arch.

**Figure 4:**
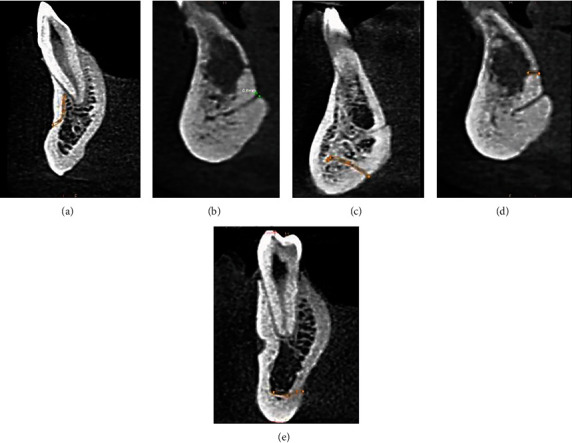
Cross-sectional views of the (a) labial canal and corresponding foramina, (b) accessory canal and corresponding foramina superior to the genial tubercle, (c) accessory canal and corresponding foramina inferior to the genial tubercle, (d) median lingual accessory canal and corresponding foramina measured in diameter at the exit of the canal, and (e) accessory canal and corresponding foramina lateral to the genial tubercle.

Among 50 CBCT scans studied, all the scans had a minimum of three and a maximum of 11 canals. The mandible was divided into anterior and posterior sections taking the mental foramina as a reference point for ease of study. The anterior section was defined as “part of the mandible between two mental foramina,” while the posterior section was defined as “part of the body of the mandible posterior to the mental foramina up to the retromolar fossa region.” So one anterior section and two posterior sections, right and left, were obtained per scan. Thus, the canals in the posterior part were further divided into right and left posterior accessory canals. The anterior section was subdivided into labial and lingual sections. The labial section was divided into right and left labial accessory canals, and the lingual canals were subdivided into medial and lateral canals. The medial canals were further divided into superior and inferior, whereas the lateral canals were divided into right and left lateral subsections taking the genial tubercles as a reference (Figure 2). They were named as accessory canals superior to genial tubercles (SGTC), accessory canals inferior to genial tubercles (IGTC), right lateral to genial tubercles canals (RLGTC), and left lateral to genial tubercles canals (LLGTC), and a schematic presentation is illustrated in Figure 3. The canals arising in the midline of the mandible were named median lingual canals. The measurements were done using the measurement tool in the CS 3D software. The cross-sectional views of all accessory canals are shown in Figure 4. A total of 243 accessory canals and 245 accessory foramina were found, out of which 53% accessory canals were found in males while 47% were seen in females. Three accessory canals were present in 20% scans, four were seen in 30%, 5 were seen in 22%, 6 were seen in 20%, 9 were seen in 6%, and a maximum of 11 canals were seen in 2% scans, respectively (Table 1). About 89% (216) accessory canals were seen in the anterior region, while only 11% (27) were seen in the posterior region, which is statistically significant (*p* < 0.05). In the anterior region, out of 89%, around 76% (54% medial; 22% lateral) were lingual canals, 13% were labial canals, and the findings were statistically significant (Table 2).

The mean diameter of accessory canals in males was 3.624 ± 1.58 mm, and in females, it was 3.292 ± 0.98 mm (Table 3). The mean length of accessory canals in males was 4.72 ± 1.671 mm with a standard error of 0.334, and in females, it was 5.08 ± 1.579 mm with a standard error of 0.316. No statistically significant difference was found between males and females regarding the diameter and length of the accessory canals. On applying the *t*-test for equality of means, it was found that the length of the median lingual canals in males was significantly greater than that in females (*p* value 0.012). Simultaneously, there was no significant difference between males and females regarding the median lingual canals' number and diameter (Table 4). 64% of the midline lingual canals had a diameter greater than or equal to 1 mm, while only 36% had a diameter less than 1 mm, which has a statistical significance.

In the present study, out of 50 scans, 22% of scans showed lateral lingual canals. 65% of the total lateral lingual accessory canals were present on the right side, and only 35% were on the left side, which is statistically significant (Table 5). In 46% of males, lateral lingual canals were present, while 54% of females showed lateral canals (Table 5). However, no significant difference was observed between males and females in this regard. Out of the total 54 lateral lingual canals found in the present study, 81% (44) were unilateral, while 19% (10) were bilateral. The unilateral canal refers to a lateral lingual canal on one side of the mandible, either right or left, whereas the bilateral canals refer to the lateral lingual canals on both sides (right and left) of the mandible. Unilateral canals were seen in 18% of the subjects, and bilateral canals were seen in only 4% of the subjects. However, no lateral lingual canals were seen in about 78% of the subjects (Table 6). Out of the total 54 lateral lingual canals found in the present study, 81% (44) were unilateral, while 19% (10) were bilateral.

## 4. Discussion

Surgical intervention is one of the most critical aspects of the treatment procedures in dentistry. Extreme importance and priority should be given for preoperative clinical and radiographic examinations to dodge intraoperative and postoperative complications, functional impairment, and surgical stress [6]. As the result of an arterial trauma induced by improper instrumentation, there may be a chance of massive internal bleeding in the floor of the mouth, usually due to the lingual cortical plate's perforation. The intensifying lingual, sublingual, submandibular, and submental hematomas tend to displace the tongue and floor of the mouth, resulting in airway obstruction [7]. To reduce the possibility of such significant complications, appropriate evidence on the microvasculature at the surgery site is paramount. Nerve injury may occur during local anesthesia administration, bone preparation, or implant placement. The common complications associated with nerve injury during implant surgery include altered sensation and persistent pain after implant placement, which may be neuropathic and does not respond well to conventional analgesics and opioids [8, 9]. Particularly in the presence of accessory canals and due to postextractive ridge resorption, bone quantity could not be sufficient to place an adequate implant without causing nerve injuries. To prevent this risk, a few authors proposed using ridge preservation techniques for both anterior and posterior sites [11, 12]. Delivering profound and proper anesthesia is imperative for the success of any surgical procedure, and hence, it is of utmost importance to have a comprehensive knowledge of the appropriate anatomy of accessory canals. The radiographic examination helps assess these accessory canals' location, position, relation, and dimensions. CBCT is a medical imaging technique dedicated to imaging the oral and maxillofacial region, which heralds a true paradigm shift from 2D to a 3D world. It provides fast and accurate visualization of bony anatomical structures at a lower cost and absorbed dose than the conventional CT [12]. Since very few studies have been reported in the literature that has used CBCT to assess mandibular accessory canals, CBCT was used in the present study. Also, various classifications are mentioned in the literature for the morphology of accessory canals. Most of them are about the root canals [13], and others are vague and unreliable.

Aps [1] has observed accessory canals in 94.6% of the scans, whereas canals were not evident in 5.4% of the scans. Similarly, an Italian study [14] found accessory canals in 90.35% of the scans and no canals in 9.65%. However, in the present study, based on the finer, 0.180 mm slice thickness of the CBCT images, all the scans analyzed showed at least one lingual perforating accessory canal in the mandibular arch. These findings were in agreement with earlier studies from Austria [15], South Africa [16], and Taiwan [17]. The mean number of accessory canals was 3.8 in the study by Aps [1], while in the present study, it was 4.5. The accessory canals present were 3-11 per scan in the present study. Similarly, Tepper et al. [15] have found 1-5 canals per scan; Aps [1] has found 0–11 canals per scan. Elani et al. [18] and Liang et al. [19] have reported 1-4 canals per scan in their respective studies. In the present study, three accessory canals were present in 20% of scans, 4 in 30%, 5 in 22%, 6 in 20%, and 9 in 6%, and a maximum of 11 canals were seen in 2% of scans observed in our subjects. Aps [1] has reported 2-6 canals in 81% of cases, 1-5 canals in 71.6% of cases, and >5 canals in 23% of cases. However, Tepper et al. [15], Wang et al. [17], Elani et al. [18], and Liang et al. [19] have found two accessory canals in most of the scans in their respective studies. In contrast, an Italian study [14] found a single accessory canal (44.7%) commonly in their 115 scans. The findings in the present study were in contrast with all the published studies. The number of canals in the anterior region was significantly greater than that in the posterior region (*p* < 0.01). Similar findings were reported in the studies done by Scaravilli et al. [14], Tepper et al. [15], and Wang et al. [17]. In the present study, out of 89% of the anterior accessory canals, 76% of canals comprise lingual canals, and 13% comprise labial canals, which is statistically significant (*p* < 0.001). None of the studies published in the literature has mentioned labial accessory canals in detail. This definition establishes the posterior limit of the analysis of anatomical variations in the study. However, posterior teeth are often missing, and their absence requires a treatment direct distally to the last present tooth; indeed, dental implants are mainly used to replace posterior teeth [20].

Kilic et al. [21] analyzed 200 CT images and found 236 median lingual canals and 159 lateral lingual canals. This difference between the number of lingual canals in the midline and lateral regions was significant (*p* < 0.001). A similar finding was observed in the present study, where 130 medial lingual canals and 54 lateral lingual canals were found in 50 scans, which was statistically significant. A study from Belgium [16] reported a considerable gender effect on the occurrence of single, double, or more canals. The authors found that males more commonly had double canals while women tended to have single canals in the mandibular midline region (*p* < 0.05). In the present case, a more significant number of canals were seen in males compared to females. Also, the maximum number of accessory canals was inferior to the present study's genial tubercles. However, Elani et al. [18] found the maximum number of canals superior to the genial tubercles. In the study by Gahleitner et al. [22], the lingual canals' mean diameter was found to be 0.7 ± 0.3 mm in the midline and 0.6 ± 0.2 mm in both premolar regions of the mandible. P. Jaju and S. Jaju [23] observed the mean diameter of the lingual vascular canals to be 0.31 ± 0.70 mm in 75 Indian subjects. The findings from the present study were in agreement with this Indian study.

In the present study, about 22% lateral lingual canals were present, out of which 65% canals were seen on the right side while 35% were seen on the left side. No lateral canals were observed in about 78% of the scans, while unilateral canals were noticed in 18% of the canals, and only 4% of the scans showed bilateral canals. As reported in earlier studies, the frequency of lateral lingual canals ranged from 30% to 70% [15, 24]. A Japanese study [25] found 55% bilateral lingual canals, whereas Przystańska and Bruska [26] reported 36% bilateral lingual canals, and Patil et al. [27] observed the occurrence of 34.1% bilateral lingual canals. Following these studies, bilateral lingual canals in the present study were 29.5%. Sahman et al. [28] reported that 24.8% of the subjects in their sample showed lateral lingual canals, which is similar to the present study. Out of these lateral lingual canals, 70.2% were unilateral and 29.8% were bilateral, and these findings were in agreement with the present study. The difference between the diameters of the sides of the lateral lingual canals was not statistically significant (*p* > 0.05). The mean diameter of the lateral lingual canals of males was greater than that of females. However, the difference in the diameter of the lateral lingual canals between males and females was not statistically significant (*p* > 0.05). These findings are in agreement with the present study.

The accessory canal diameter is essential since it directly correlates with the diameter of the vessels passing through it [22]. Hence, the potential risk of hemorrhage increases with the increasing diameter of these canals. Most of the studies reported in the literature state that the maximum number of canals is present in the mandible midline, as seen in the present study. In the present study, the median lingual canal diameter was >1 mm in 64% of the canals, while in 36% of the canals, it was <1 mm. Small blood vessels with a diameter of <1 mm are rarely problematic, even if the drilling procedures for dental implant insertion resect these blood vessels. Resection of these larger blood vessels causes excessive hemorrhage secondary to arterial injury or lingual cortex perforation may result, and the injured artery may prolapse into the floor of the mouth. In the worst case, bleeding into the sublingual space may produce a pseudo-Ludwig phenomenon, in which the tongue and the floor of the mouth are elevated, thus causing upper airway obstruction [17]. The majority of the lateral lingual canals have a diameter of more than 1 mm. Still, it is wise to advise CBCT imaging of the lateral lingual canals in the interforaminal region before dental implant planning because of its arterial contents.

Some authors have suggested the lingual vascular canal as a potential anatomic cause of mandible osteonecrosis in patients on bisphosphonate treatment; another report suggests that the lingual vascular canal may act as an entry point to spread tumors [29]. Other investigators have suggested alveolar ridge resorption as a risk factor for lingual cortical plate perforation in patients with an atrophic edentulous mandible during implant placement and other surgical procedures [14, 29, 30]. In the assessment of accessory canals and foramina in the mandibular arch using cone-beam computed tomography and a new classification for mandibular accessory canals, the authors aimed to evaluate the presence of accessory canals and foramina radiographically in the mandible body using CBCT, and consequently, they propose a new classification for accessory canals. Although the mandibular canal ramifications may have important clinical significance, these anatomical variants are often misunderstood and are not regularly paid attention to while performing oral surgeries. These accessory canals may contain neurovascular elements, and if they were injured, they could cause intraoperative and postoperative surgical complications [32]. Although prosthetic restorations with implants are reliable in the medium [10, 11] and long term [32], early complications during the surgical phase can occur due to accessory canals [33]. Therefore, it will be necessary to make use of a 3D imaging technique to increase accuracy, have a higher resolution, and reduce scan time and radiation dose. Furthermore, the use of CBCT also eliminates the superimposition of surrounding structures, providing additional clinically relevant information [34]. However, further studies are recommended to verify these interesting findings in all these cases. The CBCT plays a vital role in evaluating the anatomical variations in the mandibular neovascularization as it permits the visualization of the accessory canals and foramina with high resolution and accuracy. Knowledge of these structures is essential before any surgical procedure is planned, and failure to identify these anatomical variations can complicate the surgery and cause adverse consequences. Potentially, the numerous accessory canals reported in the present study could facilitate a direct pathway into the cancellous bone of the mandible.

## 5. Conclusion

The present study concludes that patients without an accessory or nutrient canals are exceptional. Ignorance of these structures may give rise to complications such as perforation of the lingual cortical bone and injury to a blood vessel during implant placement or other surgical procedures in the mandible body, especially the interforaminal region. The proposed new classification of accessory canals could be endorsed with further studies essential with a convenient sample.

## Figures and Tables

**Figure 1:**
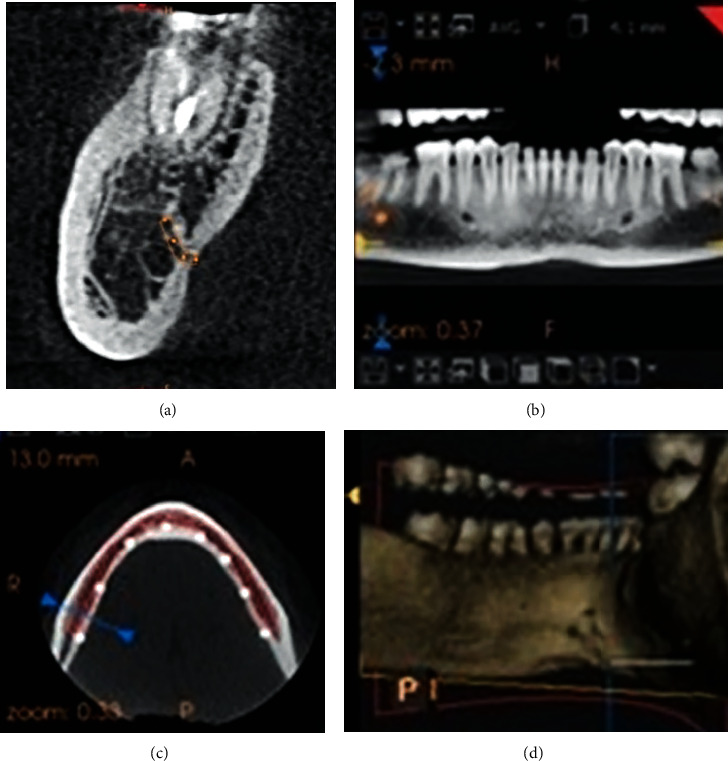
Posterior accessory canals and corresponding foramina of the right side in (a) a cross-sectional view, (b) a sagittal view, (c) an axial view, and (d) 3D reconstruction.

**Table 1:** Distribution of frequency of accessory canals in males and females.

Number of accessory canals	Gender		Total
	Female	Male	
3	7	3	10
4	7	8	15
5	6	5	11
6	3	7	10
7	0	0	0
8	0	0	0
9	2	1	3
10	0	0	0
11	0	1	1
Total (%)	115 (47.32%)	128 (52.67%)	243 (100%)

**Table 2:** Distribution of the presence of accessory canals in the body of the mandible.

Accessory canals	Anterior canals							Posterior canals		Total
	Lingual canals					Labial canals				
	Medial canals			Lateral canals				Right	Left	
	SGTC	IGTC	Median lingual	Right	Left	Right	Left			
No. (%)	15 (6)	65 (27)	50 (21)	35 (14)	19 (8)	12 (5)	20 (8)	10 (4)	17 (7)	243 (100)
Combined no. (%)	130 (54)			54 (22)		32 (13)		27 (11)		

SGT: superior to genial tubercles; IGT: inferior to genial tubercles.

**Table 3:** Diameter of accessory canals in males and females.

A5ccessory canals	Gender	*N*	Mean	Std. deviation	Std. error
SGT	Male	25	0.22	0.37	0.07
	Female	25	0.12	0.34	0.07
IGT	Male	25	0.85	0.89	0.18
	Female	25	0.87	0.69	0.14
RGT	Male	25	0.41	0.50	0.10
	Female	25	0.40	0.40	0.08
LGT	Male	25	0.36	1.03	0.21
	Female	25	0.28	0.28	0.06
RL	Male	25	0.13	0.23	0.05
	Female	25	0.13	0.23	0.05
LL	Male	25	0.17	0.43	0.08
	Female	25	0.30	0.43	0.08
RP	Male	25	0.14	0.38	0.07
	Female	25	0.11	0.36	0.07
LP	Male	25	0.34	0.34	0.07
	Female	25	0.12	0.28	0.06
Total diameter	Male	25	3.62	1.58	0.35
	Female	25	3.3	0.98	0.19

Note: SGT: superior to genial tubercles; IGT: inferior to genial tubercles; RGT: right side of genial tubercles; LGT: left side of genial tubercles; RL: right labial canals; f: left labial canals; RP: canals in right posterior section; LP: left posterior.

**Table 4:** Number, length, and diameter of median lingual canals among the genders.

Variable	Gender	Mean	Std. deviation	Std. error mean
Total number	Male	1.04	0.20	0.04
	Female	1.00	0.00	0.0
Length	Male	12.81	2.64	0.53
	Female	11.28	1.29	0.26
Diameter	Male	1.01	0.18	0.04
	Female	0.98	0.13	0.03

**Table 5:** Distribution of lateral lingual accessory canals in males and females.

Accessory canals	Male	Female	Total
Right side	18 (51%)	17 (49%)	35 (65%)
Left side	6 (31%)	13 (69%)	19 (35%)
Total	24 (46%)	30 (54%)	54 (100%)

**Table 6:** Unilateral and bilateral distribution lateral lingual accessory canals in males and females.

Accessory canals	Males	Females	Total (%)
No. of canals	129 (68%)	60 (32%)	189 (78%)
Unilateral	21 (48%)	23 (52%)	44 (18%)
Bilateral	3 (30%)	7 (70%)	10 (4%)

## Data Availability

The data that support the findings of this study are available from the corresponding authors upon reasonable request.
